# Nonalcoholic Fatty Liver Disease: Could It Be the Next Medical Tsunami?

**DOI:** 10.7759/cureus.23806

**Published:** 2022-04-04

**Authors:** Priyanka Bhandari, Amit Sapra, Mohitkumar S Ajmeri, Christine E Albers, Devanshika Sapra

**Affiliations:** 1 Family Medicine, Southern Illinois University School of Medicine, Springfield, USA; 2 Family and Community Medicine, Southern Illinois University School of Medicine, Springfield, USA; 3 Family and Community Medicine, Southern Illinois University, Springfield, USA; 4 Neuroscience, Saint Louis University, St. Louis, USA

**Keywords:** nafld vs nash, nafld symptoms, nafld treatment, nafld causes, nafld diagnosis, reverse your fatty liver, nafld pathophysiology, risk factors for nash, how is nafld caused, how common is nash

## Abstract

Nonalcoholic fatty liver disease (NAFLD) is a rapidly increasing cause of chronic liver disease with excess fat deposition in the liver, without an identifiable cause. NAFLD's benign form is called nonalcoholic fatty liver (NAFL), which can progress to nonalcoholic steatohepatitis (NASH) with or without fibrosis. Over time, NASH can progress to cirrhosis and eventually hepatocellular carcinoma (HCC) or progress to HCC without cirrhosis. Its incidence and prevalence are increasing to epidemic proportions, making it the most common cause of chronic liver disease in the western world. This review article attempts to understand the epidemiology, pathophysiology, evaluation, and management, and, most importantly, to generate awareness of this disease process.

## Introduction and background

Nonalcoholic fatty liver disease (NAFLD) is caused by the accumulation of fat cells in the liver. Its rate has been on the rise globally over the last few decades, paralleling the rise in obesity worldwide. NAFLD includes both the benign condition of fatty liver or steatosis without inflammation known as nonalcoholic fatty liver (NAFL) and the progressive liver condition that leads to lipotoxicity and inflammatory damage known as nonalcoholic steatohepatitis (NASH) [1]. Unlike NAFL, NASH has a high risk of progressing to cirrhosis, liver failure, and liver cancer. NASH has been stated as the major cause of chronic liver diseases (CLDs) in this decade, and NASH-related cirrhosis will become the most frequent indication for liver transplants in the upcoming years [1]. It has also been reported as the most common cause of CLD in the pediatric population [2]. NAFLD is closely associated with obesity, metabolic syndrome, insulin resistance, and type 2 diabetes mellitus [3]. With the rise in epidemic proportions, it is imperative that we must take an “all hands on deck” approach to take care of this growing menace.

## Review

What is NAFLD?

NAFLD is an umbrella term for a range of conditions, defined by the presence of fat accumulation in the hepatic parenchyma and the presence or absence of inflammation. NAFL, the most benign form of NAFLD, is defined as an accumulation of fat (≥5%) in the hepatic parenchyma in which there is no evidence of any inflammation or cellular damage [4]. On the other hand, NASH is defined by the presence of fat (≥5%) and the presence of inflammation and/or ballooning degeneration of hepatocytes with or without Mallory bodies [4]. It is important to understand that to diagnose NAFLD, all other causes of CLD, including alcoholism-induced hepatitis C, have been excluded, as NAFLD is associated with increased body mass index (BMI), insulin resistance, metabolic syndrome, and diabetes mellitus [3].

Difference between NAFL and NASH

Patients with NAFL have a very slow progression (in general) to fibrosis. Unlike NAFL, NASH has a high risk of progressing to cirrhosis, liver failure, and liver cancer, though it can progress directly to cancer without going through cirrhosis. Patients with NASH can exhibit histological progression and develop fibrosis (37‐41%) and cirrhosis (5%), and they have higher liver‐specific mortality and higher cardiovascular mortality due to risk factors associated with metabolic syndrome and have decreased survival [4]. NASH has been stated as the major cause of CLD in this decade, and NASH-related cirrhosis will become the most frequent indication for liver transplants in the upcoming years [1]. It has also been reported as the most common cause of CLD in the pediatric population [2].

Prevalence of NAFLD

There has been a progressive rise in NAFLD rates, parallel to the rising BMI, metabolic syndrome, and insulin resistance states. About 50-70% of patients diagnosed with diabetes mellitus also have NAFLD. Researchers estimate that the global prevalence of NAFLD is around 25% in the adult population but has wide variations all across the globe [5]. While countries in the Middle East and South America top the prevalence rates at around 30%, prevalence rates in North America and Europe have been reported to be around 24% [5]. Prevalence in Africa has been around 13%, whereas prevalence rates in Asia have shown a wide variation from 5% to 51% [5].

**Figure 1:**
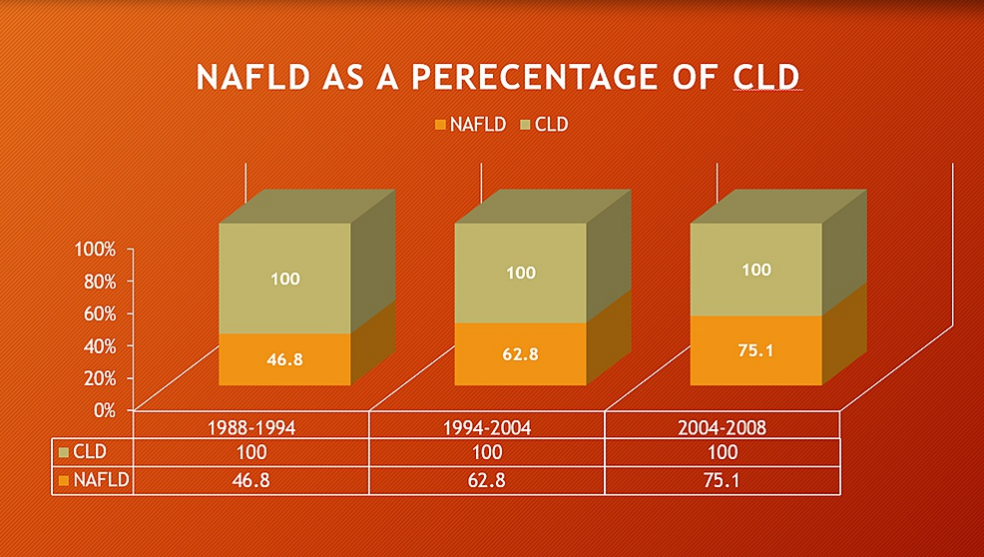
Prevalence of NAFLD as a percentage of total cases of CLD over time. The above bar graph depicts the progressive rise in the prevalence of nonalcoholic fatty liver disease (NAFLD) as a cause of chronic liver disease (CLD) in the USA in the last few decades [6].

Figure 1 shows that the prevalence of NAFLD has been increasing over time in the USA, as seen by reports from the National Health and Nutrition Examination Survey (NHANES). NAFLD accounted for 46.8% of all cases of CLD between the years 1988 and 1994. This number rose to 62.84% between the years 1994 and 2004. The trend continued with NAFLD accounting for almost 75% of cases between 2005 and 2008 [6].

Researchers have reported NAFLD prevalence to be from 3% to 12% in the entire pediatric population but could go up to as high as 70-80% in children considered obese [2]. Prevalence data by race show NAFLD to be most prevalent in patients of Hispanic origin [2].

Causes of NAFLD

**Figure 2:**
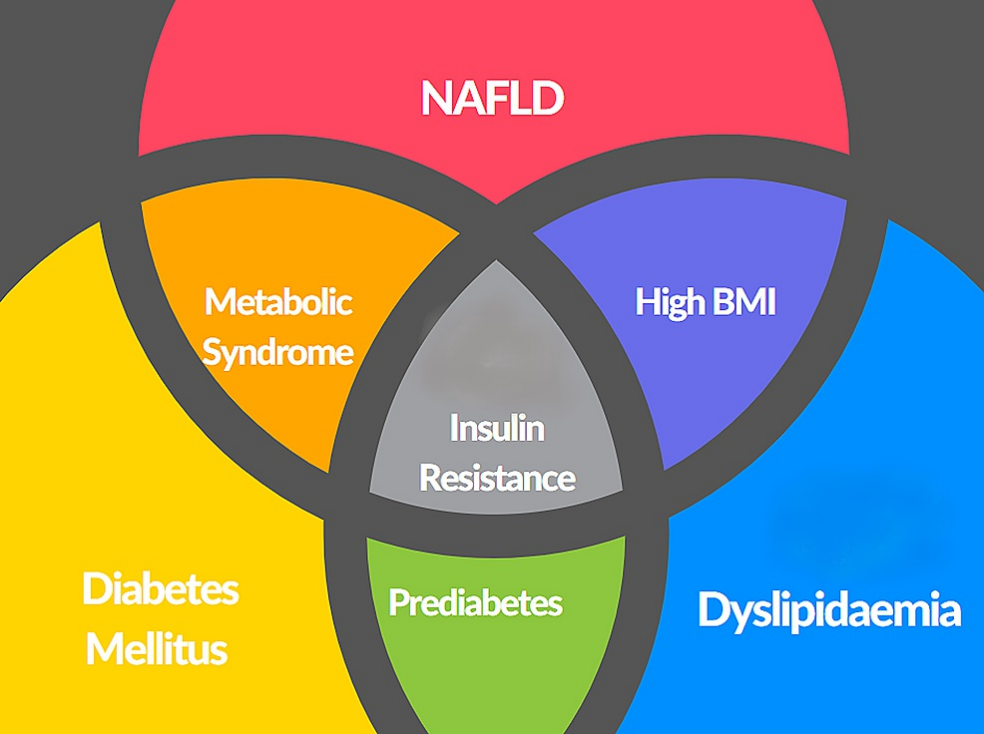
Associations of nonalcoholic fatty liver disease (NAFLD).

The pathogenesis of NAFLD is not completely clear. The underlying insulin resistance and consequent hyperinsulinemia seem to be the main culprit driving the development of NAFLD [7]. The most common causes of NAFLD are insulin resistance, high BMI, metabolic syndrome, and type 2 diabetes mellitus. The metabolic syndrome, also called syndrome X in the past, is associated with increased waist circumference, increased blood pressure, increased triglycerides, increased blood sugars, and low levels of high-density lipoproteins (Figure 2). The development of insulin resistance and subsequent development of metabolic syndrome is central to the pathogenesis of NAFLD. Not surprisingly then, the common associations include high BMI, sedentary lifestyle, type 2 diabetes mellitus, hypertension and dyslipidemia, polycystic ovarian syndrome, obstructive sleep apnea, and hypothyroidism, among others. Medications that can increase the risk of developing metabolic syndrome include second-generation antipsychotics, antiretroviral medications, and steroid intake. Other factors include genetics and race. Studies have shown that the Asian population can develop metabolic syndrome and subsequently NAFLD even with lower BMI. Genetic factors that increase insulin resistance might have a role to play as well [7].

**Figure 3:**
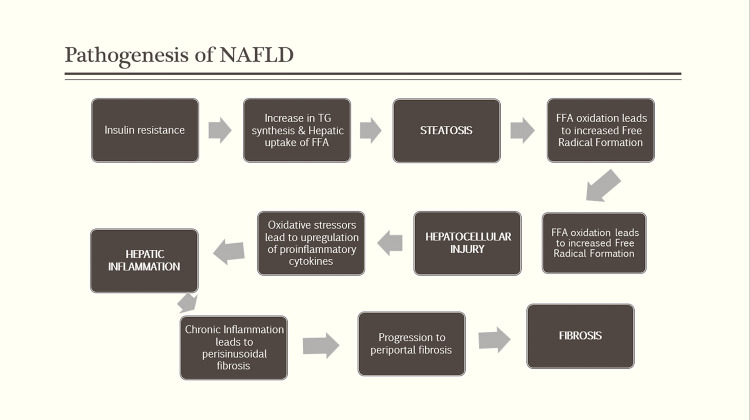
Pathogenesis of nonalcoholic fatty liver disease (NAFLD) Adapted from [12,13]. TG: triglycerides; FFA: free fatty acid.

Hepatic fat accumulation and insulin resistance, typically in the context of obesity, play an important role. Other pathophysiological factors, including oxidative stress, cytokine‐mediated injury, and increased fibrogenesis, may contribute to the development of significant liver disease [8]. Moreover, hepatic steatosis is due to excess accumulation of toxic lipids in the liver, including triglycerides, free fatty acids (FFAs), ceramides, and free cholesterol [9]. This can occur from the excessive importation of FFA from adipose tissue, from diminished hepatic export of FFA (secondary to reduced synthesis or secretion of very-low-density lipoprotein), or impaired beta-oxidation of FFA [10]. FFAs are inducers of several cytochrome p-450 microsomal lipoxygenases, capable of producing hepatotoxic free oxygen radical species [11]. Furthermore, the shift to FFA beta-oxidation in the setting of pre-existing defects in mitochondrial oxidative phosphorylation may result in even more free radical formation and subsequently hepatocellular injury and fibrosis [12]. Chronic hepatocellular inflammation and injury lead to perisinusoidal fibrosis with activation of lobular stellate cells. Portal fibrosis is commonly a feature of progressive disease. It stems from activating a secondary replicative pathway involving hepatic progenitor cells. Hepatic progenitor cells appear to proliferate in the setting of primary replicative senescence from chronic hepatocyte injury. A ductular reaction ensues, leading to periportal fibrogenesis. An increase in the ductular reaction is correlated with the grade of NASH activity, the degree of fibrosis, and the extent of primary hepatocyte replicative arrest, which in turn correlated with insulin resistance (Figure 3) [12,13].

Can lean individuals develop NAFLD?

**Figure 4:**
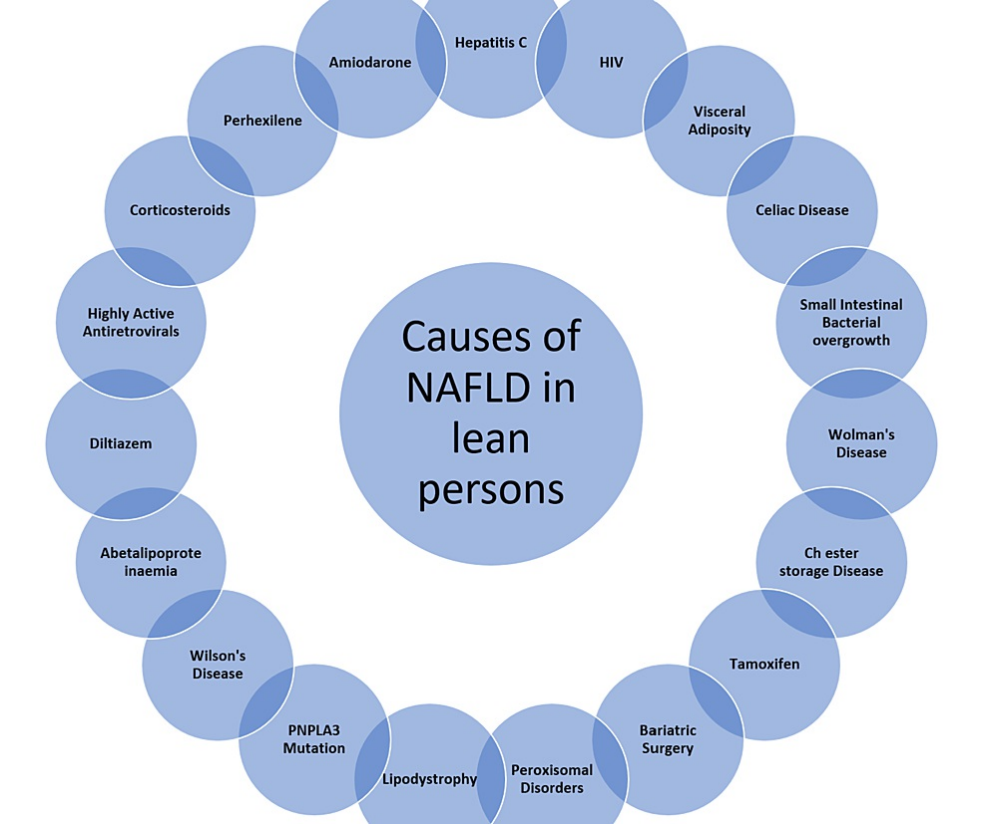
Causes of nonalcoholic fatty liver disease (NAFLD) in lean individuals. PNPLA3: patatin-like phospholipase domain-containing protein 3.

Of individuals with a lean habitus, 7-20% can have NAFLD [14]. Even in a lean patient, hyperinsulinemia can cause NAFLD development [7]. As depicted in Figure 4, these people are often of Asian descent and have risk factors including insulin resistance, high body fat, high carbohydrate, and fat diet consumption [14]. Many medications are a risk factor for the disease and genetic factors associated with NAFLD [14,15].

Clinical manifestations of NAFLD

Most of the patients are asymptomatic or have vague, non-specific complaints, including the right upper quadrant discomfort, fatigue, or general feeling of being unwell. Often, the patients have been informed of the diagnosis when they had imaging done for unrelated or comorbid conditions. Often the clinicians have to keep a high suspicion when evaluating patients presenting with high BMI, metabolic syndrome, or obesity. Physical exam looking for the NAFLD spectrum often presents with many nonspecific symptoms; however, most patients are asymptomatic [15]. The most common clinical sign is hepatomegaly [15]. Some other presenting symptoms are fatigue, sharp or dull right upper quadrant abdominal pain, bloating, thirst, and sleep disturbances.

Laboratory findings in NAFLD

In lab work, mildly elevated serum aminotransferases are common; however, a large percentage of NAFLD patients can have normal liver enzymes [16]. Typically, the aspartate aminotransferase (AST) to alanine aminotransferase (ALT) ratio is usually less than one [16]. In NAFLD, aminotransferase levels may be elevated two to four times over the upper limit of normal, with ALT being higher than AST, in contrast to alcoholic steatohepatitis [16]. As the disease progresses, hypoalbuminemia, thrombocytopenia, hyperbilirubinemia, and prolonged prothrombin time are also present due to hepatic synthetic dysfunction. Other blood work may also be done to rule out coexisting diseases that may damage the liver, such as hepatitis panel, iron panel, and thyroid function tests, and tests for serum ceruloplasmin and alpha antitrypsin levels should also be carried out [15]. Ruling out drug-induced liver injury is also important. Other lab work, including testing for insulin resistance by homeostasis model assessment (HOMA) calculated as a product of fasting blood glucose and fasting insulin levels, should be carried out [15].

What about other biomarkers?

The unreliability and lack of predictive ability of the transaminases have led to the emergence of various biomarkers [17]. These can be divided into those used for NAFL, NASH, or fibrosis [17]. Besides providing helpful insight into clinical practice, these biomarkers (Tables 1, 2) can collect data in retrospective research studies [17].

**Table 1:** Biomarkers for NAFL. The various biomarkers, which are a conglomeration of readily available laboratory results, help establish the diagnosis of NAFL [17]. NAFL: nonalcoholic fatty liver; NAFLD: nonalcoholic fatty liver disease; ALT: alanine aminotransferase; GGT: gamma-glutamyl transpeptidase; TGs: triglycerides; AST: aspartate aminotransferase; DM: diabetes mellitus; NASH: nonalcoholic steatohepatitis; HAIR: hypertension, ALT, insulin resistance.

Test name	Markers	Purpose of the test	AUROC (area under the curve)
Steatosis test	ALT, alpha -2 macroglobulin, Apo-A1 haptoglobin, total bilirubin, GGT, TGs, sugar, age, sex, and BMI	Diagnosis of NAFL	0.79
Liver fat score	AST, insulin, metabolic syndrome, DM type 2, and AST/ALT	Diagnosis of NAFL	0.87
Hepatic steatosis index	ALT, AST, age, BMl, and sex	Diagnosis of NAFL	0.812
Plasma cytokeratin (CK)-18	CK-18 (emerging as one of the promising biomarkers for the noninvasive detection of NASH)	Distinguishing NASH from NAFL	0.93
NASH test	Age, sex, height, weight, and serum levels of TGs, cholesterol, a2-macroglobulin, apolipoprotein A1, haptoglobin, GGT, ALT, AST, and total bilirubin	Distinguishing NASH from NAFL	0.79
HAIR	Hypertension, ALT, and insulin resistance	Distinguishing NASH from NAFLD in bariatric surgery patients	0.9
NASH clinic scoring system	Hypertension, DM type 2, AST > 27 IU/L, ALT > 27 IU/L, sleep apnea, and nonblack race	Distinguishing NASH from NAFLD in bariatric surgery patients	0.8

**Table 2:** Biomarkers for fibrosis. The various laboratory parameters available for the patient can give insight into the chances of developing fibrosis in patients with NAFL [17]. NAFL: nonalcoholic fatty liver; NAFLD: nonalcoholic fatty liver disease; FIB-4: fibrosis-4; APRI: aspartate aminotransferase (AST) to platelet ratio index; ALT: alanine aminotransferase; GGT: gamma-glutamyl transpeptidase; AST: aspartate aminotransferase; DM: diabetes mellitus; BARD: BMI, AST/ALT ratio, diabetes.

Test name	Markers	Purpose of the test	AUROC (area under the curve)
NAFLD fibrosis score	Age, BMI, hyperglycemia, platelet count, albumin, and AST/ALT ratio	Distinguishing fibrosis	0.85
Fibro meter	Glucose, AST, age, weight, ferritin, ALT, and platelet count	Distinguishing fibrosis	0.936
Fibro test	a2-macroglobulin, haptoglobin, apolipoprotein A1, GGT, total bilirubin, and ALT	Distinguishing fibrosis	0.86
BARD	BMI, AST/ALT ratio, and presence of DM	Distinguishing fibrosis	0.81
FIB-4	Age, AST, ALT, and platelet counts	Distinguishing fibrosis	0.802
APRI	AST/platelet x 100	Distinguishing fibrosis	0.86 for significant fibrosis, 0.861 for severe fibrosis, and 0.842 for cirrhosis

Due to its wide availability, less cost, moderate accuracy, and ability to screen for hepatic carcinoma, abdominal ultrasound is routinely ordered to diagnose NAFLD in everyday clinical practice [17]. It is, however, operator-dependent and is not very sensitive with a BMI above 40 kg/m2. The amplitude of ultrasound waves is dampened in a fatty liver and is currently being extensively used in controlled attenuation parameter (CAP) by FibroScan® (Echosens, Paris, France) [17]. More recently, proton magnetic resonance spectrometry (H-MRS), magnetic resonance density fat fraction, and magnetic elastography have emerged as the most accurate and most sensitive noninvasive tests for measuring the amount of liver steatosis [17,18]. Noninvasive tests described above are useful, but liver biopsy is considered the definitive diagnostic tool due to the superior quality, reference standard, standardized interpretation, and pharmacological treatment [18]. The American Association for the Study of Liver Diseases (AASLD) and the American College of Gastroenterology consider liver biopsy as the gold standard test for measuring fibrosis [18].

Management of NAFLD

Lifestyle Interventions

Lifestyle changes remain the first-line treatment for patients with nonalcoholic liver disease. Weight loss is the most important therapy for these patients and has been shown to improve liver function tests and improve the histological picture while increasing insulin sensitivity [19]. Decreasing calorie intake through a very low-calorie diet (VLCD), low-calorie diet (LCD), and low-fat diet has been shown to improve lower alanine transferase levels (ALT), lower glucose levels, improve insulin sensitivity, and improve NAFLD [19]. The dietary composition has also been shown to play a role, and an isocaloric Mediterranean diet and the dietary approach to stop hypertension (DASH) have shown similar benefits [19]. High-calorie sugar-sweetened beverages, especially rich in fructose, can stimulate lipogenesis in the liver and should be avoided [20]. Weight loss of about five percent in cases of NAFLD improves hepatic steatosis and associated inflammation [20].

Physical exercise has been shown to help maintain weight loss and reduce risk factors like dyslipidemia, insulin resistance, metabolic syndrome, and decreased liver adiposity [19]. Exercise is associated with decreased hepatic fat even in the absence of weight loss [19,20]. One of the benefits of aerobic exercise is that it decreases the cardiovascular risks in these cases, contributing to almost 40% mortality versus about 10% mortality from liver-related causes. Resistance exercise helps in improving muscular strength as well as muscle mass, besides helping in improving metabolic control. Research has shown it can cause up to a 13% reduction in liver fat [20].

There is great synergy between the underlying pathophysiology causing liver damage due to alcohol usage and nonalcoholic liver disease [21]. Moderate consumption of alcohol can be poorly defined even among providers and can increase the risk of miscommunication with the patient and lead to nonadherence [21]. There is also the added detrimental effect of alcohol leading to hepatocellular carcinoma in patients.

Pharmacological Management

Insulin sensitizing agents: The mainstay of pharmacological therapy for NAFLD revolves around insulin-sensitizing medication. Metformin has a role in decreasing insulin resistance and improving liver enzymes but does not make any significant changes at the histological level [22]. Thiazolidinediones are peroxisome proliferator-activated receptor-gamma agonists used in type 2 diabetes that help differentiate the fat cells into smaller insulin-sensitive fat cells and help resolve NAFLD. When used over a period of time, they help decrease the underlying decreasing fat content of the hepatocytes and decrease inflammation and fibrosis [22]. This action is thought to be secondary to improving fat metabolism and its anti-inflammatory effect [22]. Glucagon‐like peptide‐1 agonists help in improving glucose homeostasis as well as helping in losing weight. Initial trials have shown promising results with weight loss, and a decrease in liver enzymes, though further trials are awaited [22]. Sodium-glucose co‐transporter 2 inhibitors, while preventing the reabsorption of glucose in the kidney, also prevent de novo hepatic lipogenesis. Recent trials have shown only a modest reduction in fat fraction, though the effect on the histology is still under investigation [22].

Vitamin E: Being part of the cell membrane, vitamin E protects against oxidative damage by free radicals and prevents injury to the liver by blocking intrinsic apoptotic pathways and protecting against mitochondrial activity. Vitamin E at 800 IU/day has also been shown to be of benefit in nondiabetic adult and pediatric populations, but data are lacking in diabetic patients [22,23].

Bile acid signaling: Obeticholic acid (OCA) is a farnesoid X receptor (FXR) agonist, a bile acid receptor, that leads to improved hepatic and peripheral glucose metabolism. It has been used extensively for the treatment of NAFLD. It also has an inhibitory effect on liver lipogenesis and exerts an anti-inflammatory effect while decreasing fibrosis [22]. Trials with a newer agent called NGM282, a humanized fibroblast growth factor (FGF)-19 analog, have shown similar agonist activation of the FXR [22].

Lipid altering medications: NAFLD is associated with other metabolic dysfunctions along with hyperlipidemia. Both hypercholesterolemia and hypertriglyceridemia are prevalent, and hence lipid-lowering agents benefit these patients. Inhibition of stearoyl-CoA desaturase (SCD) enzyme, the rate-limiting step in synthesizing monosaturated fatty acids, has also been shown to improve steatosis and insulin sensitivity [23]. Despite dyslipidemia being commonly associated with NAFLD, statins have been underutilized in this condition. Studies in Europe have shown that statins are protective of NAFLD [23]. These lipid-lowering agents should be offered to patients with NAFLD since they are also at a high risk of developing cardiovascular morbidity and mortality. Triglyceride lowering agent gemfibrozil has shown improvement in levels of alanine transaminases in patients with NAFLD. Ezetimibe, a lipid-lowering drug that works by decreasing the reabsorption of intestinal lipids and decreasing tumor necrosis factor-alpha levels, is also undergoing evaluation [24].

Ursodeoxycholic acid: Ursodeoxycholic acid (UDCA) has been shown to have a protective effect on the liver. It has been studied in various trials for the treatment of NAFLD. Currently, UDCA is being used as part of a combined drug regimen to treat NAFLD [24].

Newer agents: Newer agents like resmetirom (thyroid hormone receptor-beta selective agonist), cenicriviroc (chemokine receptor 2 and 5 inhibitors), emricasan (pan-caspase inhibitor), simtuzumab (monoclonal antibody against lysyl oxidase-like 2) as well as selonsertib (apoptosis signal-regulating kinase inhibitor) and FGF-21 are currently undergoing trials for their therapeutic benefit in NAFLD [25,26].

Surgical Interventions

Bariatric surgery: Bariatric surgery can cause weight loss and improve the metabolic dysfunction of the patient. Many studies have shown the benefit of bariatric surgery in patients with NAFLD. A systematic review by Lee et al. (2019) reported biopsy-confirmed resolution of steatosis, inflammation, ballooning degeneration, and fibrosis of NAFLD in obese patients; however, some patients still developed new or worsening features [27]. Bariatric surgery does carry perioperative risks, including an increased risk of mortality in patients with cirrhosis, especially in decompensated cirrhosis [28]. The AASLD and the European Association for the Study of the Liver (EASL) do mention the role of bariatric surgery in patients nonresponsive to lifestyle changes and nonsurgical options [28]. The Italian Association for the Study of the Liver (AISF) and the National Institute for Health and Care Excellence (NICE) do not mention the role of bariatric surgery. The Asia-Pacific limits its role in patients with class II obesity (BMI > 32.5 kg/m2 in Asians and 35 kg/m2 in Caucasians) [28].

Liver transplant: Cirrhosis associated with NAFLD is one of the most common reasons for liver transplantation. Due to their associated comorbidities, including obesity, metabolic syndrome, and diabetes, these patients are at higher risk of peri- and post-operative complications [28]. Apart from the AISF and the NICE guidelines, most other guidelines mention the role of liver transplants in these patients with end-stage liver disease [28].

## Conclusions

NAFLD is now the most common cause of CLD in the western world and the most common cause of liver disease in children. Its incidence and prevalence are increasing to epidemic proportions. Besides increasing the risk of developing cirrhosis and liver failure, NAFLD also increases the risk of hepatocellular cancer (HCC), one of the leading causes of obesity-related cancer deaths in the USA. It is no wonder that this exponential rise in the prevalence of NAFLD in both the pediatric and adult population has led to its comparison as “The Next Medical Tsunami.” As NAFLD cases continue to rise, it has become imperative than at any other time in the history of medicine that awareness about the disease, its diagnosis, and management are given the highest importance. The role of an inter-professional team consisting of the primary care physician, hepatologist, nutritionist, endocrinologist, and bariatric surgeon is highly needed to optimize their goals and ensure the best possible outcomes for the patients. Education through mass media about this rising epidemic needs to be propagated to the public, with the highest emphasis on primordial and primary prevention of the disease. We need to gather all our resources to fight this fast-approaching “Tsunami.”
